# Supplementary material for: Institutional differences in USMLE Step 1 and 2 CK performance: Cross-sectional study of 89 US allopathic medical schools
Source: PLoS One. 2019 Nov 4;14(11):e0224675. doi: 10.1371/journal.pone.0224675 (PMC6827894; doi:10.1371/journal.pone.0224675)
Supplement: Attachment — Submitted filename: PLOS ONE Review.docx [file pone.0224675.s002.docx]

Review of Institutional Differences in USMLE Performance: Cross sectional study of 89 US allopathic medical schools (PONE-D-19-20613

- What are the main claims of the paper and how significant are they for the discipline? The authors show that institutional mean GPA and mean MCAT scores correlate strongly with USMLE performance whereas student body demographic and other institutional variables were weak or insignificant when controlling for MCAT and GPA. Several schools had significant above and below predicted USMLE performance but not clear why. These results are not surprising, but to my knowledge first time I have seen such data clearly support what has long been surmised. If the authors listed the USMLE scores of the medical schools in the Table in the Supporting document, that would greatly enhance the interest of readers. After all the data are publicly accessible.
- Are the claims properly placed in the context of the previous literature? Have the authors treated the literature fairly? Yes, I believe so.
- Do the data and analyses fully support the claims? If not, what other evidence is required? I believe the data do support the claims although I am not an expert statistician.
- *PLOS ONE* encourages authors to publish detailed protocols and algorithms as supporting information online. Do any particular methods used in the manuscript warrant such treatment? If a protocol is already provided, for example for a randomized controlled trial, are there any important deviations from it? If so, have the authors explained adequately why the deviations occurred? N/A
- If the paper is considered unsuitable for publication in its present form, does the study itself show sufficient potential that the authors should be encouraged to resubmit a revised version? I believe it is suitable for publication
- Are original data deposited in appropriate repositories and accession/version numbers provided for genes, proteins, mutants, diseases, etc.?
- Are details of the methodology sufficient to allow the experiments to be reproduced? I believe so.
- Is the manuscript well organized and written clearly enough to be accessible to non-specialists? Yes, it is clearly written. The one suggestion is to clearly state in the conclusion that institutional mean GPA and mean MCAT scores correlate strongly with USMLE performance whereas student body demographic and other institutional variables were weak or insignificant when controlling for MCAT and GPA.
